# A survey of the clinical acceptability of screening for postnatal depression in depressed and non-depressed women

**DOI:** 10.1186/1471-2458-6-211

**Published:** 2006-08-17

**Authors:** Alan W Gemmill, Bronwyn Leigh, Jennifer Ericksen, Jeannette Milgrom

**Affiliations:** 1 Parent-Infant Research Institute, Department of Clinical & Health Psychology, Heidelberg Repatriation Hospital, Austin Health, 300 Waterdale Road, Heidelberg West, Vic 3081, Australia; 2 Department of Psychology, School of Behavioural Science, University of Melbourne, Vic 3010, Australia

## Abstract

**Background:**

Information on clinical acceptability is needed when making cost-utility decisions about health screening implementation. Despite being in use for two decades, most data on the clinical acceptability of the Edinburgh Postnatal Depression Scale (EPDS) come from qualitative reports, or include relatively small samples of depressed women. This study aimed to measure acceptability in a survey of a relatively large, community sample with a high representation of clinically depressed women.

**Methods:**

Using mail, telephone and face-to-face interview, 920 postnatal women were approached to take part in a survey on the acceptability of the EPDS, including 601 women who had screened positive for depression and 245 who had received DSM-IV diagnoses of depression. Acceptability was measured on a 5-point Likert scale of comfort ranging from "Not Comfortable", through "Comfortable" to "Very Comfortable".

**Results:**

The response rate was just over half for postal surveys (52%) and was 100% for telephone and face-to-face surveys (432, 21 and 26 respondents for postal, telephone and face-to-face surveys respectively) making 479 respondents in total. Of these, 81.2% indicated that screening with the EPDS had been in the range of "Comfortable" to "Very Comfortable". The other 18.8 % rated screening below the "Comfortable" point, including a small fraction (4.3%) who rated answering questions on the EPDS as "Not Comfortable" at the extreme end of the scale. Comfort was inversely related to EPDS score, but the absolute size of this effect was small. Almost all respondents (97%) felt that screening was desirable.

**Conclusion:**

The EPDS had good acceptability in this study for depressed and non-depressed women. Women's views on the desirability of postnatal depression screening appear to be largely independent of personal level of comfort with screening. These results should be useful to policy-makers and are broadly supportive of the Edinburgh Postnatal Depression Scale as a suitable tool for universal perinatal depression screening.

## Background

In judging the utility of any health screening program, a central question is whether the condition is sufficiently common and sufficiently serious to justify the costs of screening. In practice, screening is only worthwhile for conditions that are both common enough and serious enough, for which identification leads to a benefit for those screened and for which affordable, acceptable, high-accuracy screening is available.

Postnatal depression (PND) is a highly prevalent perinatal mood disorder, with estimates around 10% in most Westernised countries [1,2]. Adverse consequences are significant and potentially far-reaching for mothers, infants and fathers: yet diagnosis rates are low and most cases go untreated [3]. Non-identification may prolong depression and leave women vulnerable to ongoing parenting difficulties [4].

Cost-effectiveness modelling has shown that active interventions for depression, such as cognitive behavioural therapy or antidepressant medication, give value for money [5] and the cost-effectiveness of screening programs for depression has also been quantified successfully [6]. Despite this, the utility of screening for PND in particular remains the subject of some debate. A short, free, screening tool – the Edinburgh Postnatal Depression Scale (EPDS) – has been widely used for 20 years [7]. Whilst quantitative measurement of acceptability is common for other health screening and diagnostic tools [8-11] this has not been common for PND screening instruments. Although there is information on uptake rates in primary care [3] and on return rates in academic research [12], such statistics can tell us little about how women experience the EPDS as a screening tool: – in the same way that attendance rates at any health appointment tell us little about the discomfort experienced by patients. A handful of small, qualitative reports on acceptability in postnatal populations also exist. In one interview-based study, the majority of respondents reported feelings of relief and appreciation that health professionals were interested in their well being [13]. More recently Shakespeare, Blake and Garcia [14] reported the qualitative results of 39 face-to-face interviews investigating women's experience of completing the EPDS in General Practice settings in the United Kingdom. Twenty one of these women (54%) found this screening process "unacceptable" and issues of fear of stigma as well as a perceived inappropriateness of the screening venue were raised. If generally true, such results could suggest that a majority of women are distressed by the EPDS. Yet this rare study of screening acceptability was relatively small (*n *= 39 women in total) and the findings are un-replicated to date. There has been some study on acceptability among pregnant women (B. Leigh, unpublished manuscript) but to our knowledge, the only published study that has measured acceptability for the EPDS in a quantitative way in a sizeable population of postnatal women is that by Buist et al [15]. No existing acceptability study has included a large sample of postnatal women exhibiting elevated scores on the EPDS and/or a sizeable cohort of clinically depressed postnatal women. This is potentially important since any useful health screening instrument must have good acceptability not only in the general population that includes the much smaller target group, but specifically the acceptability must be high in that target group.

In this paper we report on the results of a survey of newly delivered mothers in Australia about acceptability of the EPDS, their experiences and comfort/discomfort in being screened with the EPDS and their views on the desirability of routine screening. We structured the survey so that women with and without elevated EPDS scores and women with formal DSM-IV diagnoses of depression [16] were all well represented. Most surveys were administered by post and we also employed a face-to-face approach among a small sub-sample in an attempt to replicate something of Shakespeare at al's (2003) methodology.

We thought this was worthwhile given that Shakespeare at al's results apparently run somewhat counter to the other existing indirect evidence on acceptability, and given the small numbers involved in that study. If different methods of survey might give different estimates of comfort/acceptability then this is important to know. For completeness, we therefore also conducted a sub-sample of surveys by telephone.

## Methods

### Questionnaires

#### Edinburgh postnatal depression scale

The EPDS comprises 10 items and measures current mood disturbance for women in the perinatal period. It was developed as a screening device to assist health professionals in detecting depressive symptoms in community samples of postnatal mothers [7]. Scores above a specified threshold are used in practice to judge the likelihood of depression and some researchers have suggested that scores above 9 indicate 'possible depression' while scores above 12 indicate 'probable depression' [17]. Complete Receiver Operating Characteristic curves (bivariate plots which illustrate the relationship of sensitivity versus specificity across the entire range of possible scores) have been determined previously for the EPDS in a large Australian sample [18]. The analysis in that study pointed to a range of "optimal" cut-offs at around scores of 12 or 13. This was similar to some previous Australian research [19]. The screening reported in this current study used a cut-off score of ≥ 12. Specificity, sensitivity and overall diagnostic accuracy at this cut-off were 60%, 90% and 83% respectively in our previous work [18].

#### Acceptability survey form

A simple, one-page form was used to gather responses to four questions as follows:

1. Women were asked if they remembered completing the EPDS (possible responses were to tick *Yes *or *No*).

2. Women were asked what completing the EPDS was like (an open ended question followed by four double-spaced lines across an A4 page in which to answer).

3. Women were asked how comfortable they felt in completing the EPDS (possible responses were arranged on a 5-point Likert scale labelled from *Not Comfortable*, through *Comfortable*, to *Very Comfortable*). This was our central measure of clinical acceptability.

4. Women were asked if they thought it was a good idea to screen new mothers for depression (possible responses were *Yes, No *and *Not sure*).

### Procedure

Between January 2002 and July 2005, 8,172 women in Victoria were screened with the EPDS at routine postnatal visits to Maternal & Child Health Centres as part of an ongoing screening and treatment program. The municipalities involved cover a wide socio-demographic range of neighbourhoods. For these purposes, EPDS scores of ≥ 12 were considered to be positive screening results and scores of <12 were considered negative. The rate of positive screening results was 11.7%. At the end of this period, a retrospective survey of acceptability was begun. This took five months to complete and produced a wide spread of times between screening and survey, thus allowing us to analyse the effect of time since screening on the perceived acceptability and desirability of screening. The acceptability survey was deliberately structured to encompass roughly equal numbers of:

1) women who screened positive and were referred to our specialist perinatal psychology clinic

2) women who screened positive and were not referred to our clinic

3) women with a negative screening result.

This structure helped to ensure a good representation of women with elevated EPDS scores and high numbers of clinically depressed women. To achieve this the survey aimed to reach:

1) all contactable women who had been referred to our clinic after screening with the EPDS (*n *= 290). NB: most referred women had initially screened positive, but 12 women who screened negative were also referred for assessment (eight were subsequently diagnosed as depressed).

2) a systematic, chronological section of those contactable women who screened positive but were not referred to us (every second such woman in order of date screened), during the same period of time (*n *= 323)

3) a systematic, chronological section of those contactable women who screened negative and were not referred to us (every twentieth such woman in order of date screened), during the same period of time (*n *= 307)

#### Postal survey

By post, we sent 873 survey forms to women who had completed the EPDS with their Maternal & Child Health Nurses and had consented to take part.

#### Telephone survey

Using the same survey form, a research assistant contacted 21 women by telephone and gathered responses to the same set of questions.

#### Face-to-face interview

Using the same survey form, 26 women were interviewed face-to-face by a psychologist, using the same set of questions.

### Statistics

We used logistic regression to identify variables affecting both response rates and the acceptability of the EPDS. Sequential modelling began with maximal models in which all putative explanatory variables were fitted simultaneously. Terms associated with non-significant changes in scaled deviance were removed sequentially until the minimal models necessary to explain the data were reached. Means and Odds Ratios are reported with 95% Confidence Intervals. Frequency distributions were analysed by Chi-squared tests.

Furthermore, a systematic cross-section comprising every fifth survey form (in date order) was used to explore women's open-ended responses about the EPDS. Coding was as follows. The comments in each transcript were examined and categorised using an emergent coding approach, following established conventions [20]. Specifically, coding of all transcripts was first completed in parallel by two independent coders, the two lists compared and any disagreements resolved by discussion. Where possible, newly examined transcripts were categorised into already existing categories. For each transcript, the overall affective tone of the content was also coded as positive, neutral or negative. We have not conducted a qualitative analysis of these data but report briefly on the categories that emerged and on the overall affective tone (positive versus neutral/negative) of responses. Statistical calculations were executed in SPSS 14.0.

### Ethical approval

The study was approved by Austin Health's Ethics Committee and all participants gave informed written consent.

## Results

### Population

In total, 479 postnatal women responded to the survey – just over half of the 920 who were approached: 873 by post, 21 by phone and 26 by face-to-face interview, including 245 women who had received a DSM-IV diagnosis of depression as a result of being screened and referred to our clinic. All had completed the EPDS with a Maternal & Child Health Nurse in Victoria prior to the acceptability survey:- the average length of time since postnatal screening with the EPDS was 58.3 weeks (95% CI 54.5–62.0). The mean EPDS score of all 920 women was 12.47 (95% CI 12.06–12.87). This reflects the deliberately high representation of women scoring above the cut-off (601 of those surveyed) who had a mean EPDS of 16.29 (95% CI, 16.00–16.58) compared to a mean of 5.26 (95% CI, 4.92–5.59) for the 319 who scored below cut-off. The average age of respondents, at the time they were screened, was 30.02 years (95 % CI, 32.6–33.43) and the average age of infants was 16.87 weeks (95% CI, 15.34–18.41).

The 21 women surveyed by telephone had a mean EPDS of 12.86 (CI 10.48–15.23) and those 26 interviewed face-to-face averaged 16.77 (CI 15.41–18.3). These latter 26 women had all been referred for formal psychological assessment because of concerns over their emotional health, including elevated EPDS scores.

### Response rate

All 47 women surveyed by telephone and in face-to-face interviews responded to the questions. Of those 873 women surveyed via post, 41 had changed address (survey forms were returned marked "*no longer at this address*"). The overall postal response rate among the remaining women was 432/832 (52%) and among these 832 women the likelihood of responding to the survey was related to EPDS screening score such that women who had higher scores were less likely to respond. Thus, postal non-respondents had significantly higher EPDS screening scores than postal respondents (13.3 versus 11.6, *p *< 0.05). Although detectably significant in the statistical sense, the slope of the underlying relationship is small so that for every 1-point increase in EPDS score, the odds of non-response rise only by around 4% (Odds Ratio 1.05, CI 1.03–1.07). There was no difference in response rates between those diagnosed with depression and those not diagnosed (χ^2 ^= 0.82, *df *= 1, *p *= 0.37). There was also a relatively small but statistically detectable effect of the time elapsed since screening, such that for every week since screening the chances of non-response rose by about 0.8% (OR 1.008, 95% CI 1.004–1.012) independent of EPDS score.

### Acceptability of the EPDS

#### Comfort level of completing the EPDS

**Figure 1:**
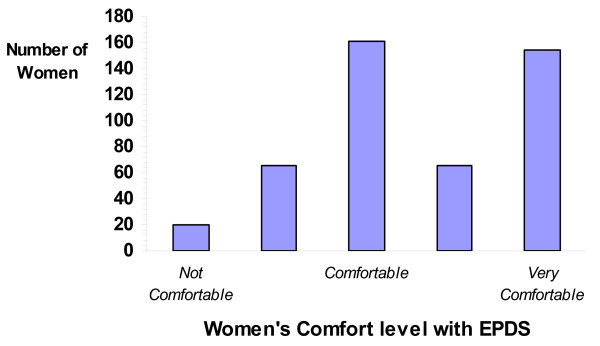
Distribution of responses on a 5-point Likert scale of comfort level in being screened with the EPDS.

To provide a central measure of clinical acceptability the survey asked "*How comfortable were you completing that questionnaire? *(the EPDS)". Of 467 respondents who answered this question, 88 (18.8%) rated their experience of completing the EPDS as less than '*Comfortable*' including 20 women (4.3%) who responded that they were '*Not Comfortable*' – the uppermost level of discomfort on the scale. The frequency distribution of responses on the 5-point Likert scale is shown in Figure 1. The distributions of responses to this question were no different for those women surveyed by post, by telephone or in face-to-face interview (χ^2^= 7.47, *df *= 8, *p *= 0.49). Next, multiple regression models were fitted, employing backward elimination of non-significant terms beginning with the maximal model. There were no significant relationships between comfort level and infant or maternal age (*p *= 0.34 and 0.99 respectively). There was a significant, negative effect of screening EPDS score such that women with higher scores tended to rate their experience of filling out the EPDS as less comfortable (slope -0.069, *p *< 0.001). However, this did not result in significantly different frequency distributions of comfort levels in women diagnosed versus those not diagnosed with depression (χ^2 ^= 7.8, *df *= 4, *p *= 0.1). There was a very small and marginally non-significant, negative effect of the time that had elapsed since screening (slope -0.003, *p *= 0.06). This suggested that, if anything, the more recently women had been screened the more comfortable they rated their experience of filling out the EPDS.

#### Desirability of screening

The survey sought women's opinions on screening by asking, *"Do you think it is a good idea to screen all new mothers for postnatal depression?" *Of 478 women who replied to this question, 462 answered affirmatively i.e. they indicated that screening was "*a good idea*". For the purpose of logistic regression, the three possible responses (*Yes, No, Not Sure*) were collapsed into a binary variable with '*Yes*' coded as 1 and with '*No*' and '*Not Sure*' both coded as 0. There were no effects of EPDS score itself (Odds Ratio = 0.97, 95% CI 0.88–1.07, *p *= 0.58) and the frequency of responses was unaffected by whether women had a DSM-IV diagnosis of depression (χ^2 ^= 0.98, *df *= 1, *p *= 0.32). There was no effect of maternal age (OR = 1.07, 95% CI 0.94–1.22, *p *= 0.32). Similarly, neither infant age at the time of screening nor the length of time elapsed since screening, exerted any effect on whether women thought screening was a good idea (ORs of 1.02 and 1.0, *p *= 0.57 and *p *= 0.82 respectively). There was no effect of the method by which women were surveyed: postal, face-to-face and telephone methods all gave the same result (χ^2^= 1.8, *df *= 2, *p *= 0.45).

Finally, the reported comfort level of respondents connected with completing the EPDS was not itself measurably related to their opinion on the desirability of universal screening (OR = 1.34, 95% CI 0.82–2.18, *p *= 0.24).

### Women's open-ended responses about the EPDS

The survey asked women *"What was it like to answer that questionnaire (the EPDS)?" *A total of 39 categories of response were identified after the emergent coding of 89 transcripts by two independent coders. Table 1 provides a summary of the commonest categories. Briefly, the broad nature of these data is as follows. Twenty four of the 39 categories occurred only once (i.e. they were each unique to a particular woman). Among the 15 that emerged for more than one individual (Table 1) a single category accounted for more than 50% of all occurrences, namely *"Screening was easy/good/fine"*.

Both independent coders and the senior author (JM) reached a consensus that 12 of the 89 transcripts examined were 'not codeable' in terms of positive, neutral or negative tone:- seven because they had no comment at all and a further five because they did not constitute a clear, coherent response relating to the actual question (e.g. one woman wrote "*Had done one 2 weeks ago*" and another wrote "*Difficult to look past how I was feeling at the time*"). Of the 77 transcripts that could be readily coded for affective tone, 61 were positive in tone, 5 were neutral and 11 were negative. Of 52 transcripts from women with a DSM-IV diagnosis 37, 4, 2 and 9 were positive, negative, neutral and not codeable respectively. Similarly, of 37 transcripts from women without a DSM-IV diagnosis 24, 7, 3 and 3 were positive, negative, neutral and not codeable. There were no systematic differences in either response rate to this question or in affective tone between women diagnosed and women not diagnosed with depression (χ^2 ^= 4.4, *df *= 3, *p *= 0.22) respectively. However, women surveyed by telephone were more likely to have a positive tone in their open-ended responses (χ^2 ^= 6.26, *df *= 2, *p *= 0.044) than those surveyed face-to-face or by post. This coding was collapsed to a binary format (positive versus neutral/negative, i.e. 61 versus 16) for the purposes of logistic regression. The overall affective tone of women's responses was not affected by maternal or infant age at time of screening, or by EPDS score (ORs 1.02, 0.99 and 1.01 respectively, *p *> 0.05 in all three cases). Only the reported level of comfort (Fig 1) was related to whether women's commentary on screening with the EPDS was itself neutral/negative versus positive (OR = 2.67, CI 1.28–5.55). Thus, the higher a woman's reported comfort level, the more likely a positive tone in her written comments on completing the EPDS.

## Discussion

Increasingly, the utility and cost-effectiveness of health interventions, including screening programs, is assessed through direct modelling of the costs (financial expenses, risks of harm etc) and the benefits (functions of screening tool accuracy, disease prevalence and the scope for disease prevention and treatment) of competing strategies [21-23]. The results of such models must still be judged in relation to so-called 'second-stage filter criteria' such as feasibility of implementation and the tolerability/acceptability of the procedure [24]. After all, even if a screening program is highly cost effective, there is no point in implementing procedures that are intolerable to patients.

This study provides quantitative data on the acceptability of the EPDS, a widespread and important health screening tool both in Australia and worldwide, in a sample that includes substantial numbers of clinically depressed women. As in a similar study with smaller numbers of depressed perinatal women [15] we found that the large majority rate their experience of being screened with the EPDS as comfortable. We have shown this result to hold true for women with elevated EPDS scores, for women with DSM-IV diagnoses of depression and for women surveyed by post, telephoned at home or interviewed face-to-face by a psychologist. Women with higher EPDS scores did tend to rate the EPDS as less acceptable (i.e. less comfortable), as one might expect intuitively, but the strength of this effect was relatively small. Furthermore, 462/479 (96.5%) respondents indicated that universal screening was a "*good idea*" and this opinion was unrelated to both EPDS score and to comfort level:- even women who did experience discomfort in screening believed that such screening was useful. This remained true even among those women who had formal DSM-IV diagnoses of depression. These are novel findings and may well be of value for professionals involved in conducting and evaluating perinatal depression screening. Nonetheless, the results raise some questions in our minds. First, could the EPDS itself be revised somehow to increase its acceptability further? Second, are there ways of administering the EPDS that help to minimise and deal with the discomfort reported by some women? How can this be done in ethical balance with the majority opinion that screening all new mothers is desirable?

When given the chance to respond in open-ended fashion, the most frequent of women's responses was that screening with the EDPS had been "*fine*", "*easy*", or "*good*". The affective tone of these responses was apparently more likely to be positive among those women surveyed by telephone and we are unsure why this should be the case. As only about 4% of respondents were surveyed by telephone this is unlikely to have affected the current results, but should be borne in mind in any future survey of women's experiences of the EPDS. Although our results indicate that in this sample, the level of acceptability for perinatal depression screening is relatively high, further studies will help to fill what is still a sizeable gap in the evidence base.

Whilst our study had a number of strengths including a relatively large sample size, the high representation of depressed women and the attempt to quantify acceptability directly, the study also had a number of limitations. First, given the relative novelty of measuring and analysing acceptability for the EPDS we proceeded by asking questions that seemed sensible and pertinent to us after reviewing how acceptability is measured and analysed for other screening procedures. Alternative methods of quantification could be explored and although we have not provided an in-depth, qualitative theme analysis of women's experiences, such an endeavour may prove valuable. In the current study we made only a simple assessment of responses in a sub-sample of women's open-ended responses. Second, we surveyed only women who had been screened via Maternal & Child Health Nurses. However, the EPDS is used in a wide range of primary health care settings and its acceptability is yet to be quantified fully in these other scenarios. For example, Shakespeare et al [14] found a low rate of acceptability among a smaller sample screened in General Practice settings in the U.K., yet we ourselves find good acceptability among Australian women in Child Health Centres. How then might acceptability vary in other countries, cultures and care settings? Does the presence and support of a Maternal and Child Health Nurse during screening enhance its acceptability? Next, on average we surveyed women about one year after their screening and this may or may not be the best time to have measured acceptability. Partly as a consequence of this timing, we had a postal response rate of just above 50% and it remains unknown if a self-selection bias could have affected our results, although given the relatively weak relationships of acceptability and opinions on screening with timing and EPDS score among the respondents we expect the broad shape of our findings to be quite robust. Finally, we were unable to collect broad demographic data in this survey and so the analysis could only compare respondents with non-respondents on a few variables (age, EPDS score etc). As such, the ability to generalise from these results is limited.

Opinion on the utility of screening for PND remains mixed. Bodies such as the American College of Obstetricians and Gynaecologists fully endorse the routine screening of postpartum women with the EPDS [25] and health bodies in other countries have adopted similar policies after due weighing of the evidence. By contrast, the United Kingdom government considers that universal screening with the EPDS does not yet meet its statutory criteria [26]. Necessarily absent from the deliberations leading to all of these policy positions were published, quantitative data on acceptability levels (virtually none were available in 2002). Yet without such data, any opinion on whether to screen or not cannot be fully informed. We hope that this study will stimulate other researchers in other countries to add to the literature on the acceptability of screening tools for PND in general, not just the EPDS.

Finally, the aim of a screening tool should not be to try and make quick diagnoses but only to better define a higher prevalence group at whom formal diagnostic procedures can be more efficiently targeted (e.g. a biopsy following a mammogram in relation to breast cancer or a formal psychiatric interview following the EPDS in the case of postnatal depression). Our current results suggest that in communities where universal postnatal screening is implemented, the EPDS has good acceptability as a tool with which to better define such a higher prevalence group.

## Conclusion

The EPDS has high rates of acceptability to respondents in this survey and this is true for both depressed and non-depressed women. Thus, the presence of the condition that is being screened for does not itself appear to impact strongly on screening acceptance in the target population. Women's views on the desirability of PND screening are largely independent of their personal level of comfort with screening and their level of depressive symptoms. These results may be useful to policy-makers when deciding on questions of universal screening implementation for perinatal depression.

## Competing interests

The author(s) declare that they have no competing interests.

## Authors' contributions

JM, JE, BL and AG conceived the study. JM, JE, BL and AG contributed to the design of the survey and the survey form. JE oversaw survey data collection and monitored the consistency of face-to-face interviews. BL collected some of the interview-based data and coded transcripts. JM resolved disagreements between coders. AG designed and executed all data analyses. AG (75%) and BL (25%) wrote a first draft of the manuscript. JM, JE, AG, and BL all edited subsequent drafts for important intellectual content and all authors agreed on the submitted version.

## Pre-publication history

The pre-publication history for this paper can be accessed here:



## Figures and Tables

**Table 1:** Common Responses. Tabulated values are the frequencies (number of occurrences in 89 transcripts examined) of common categories of response to the survey question, *"What was it like to answer that questionnaire (the EPDS)?"*

**Category**	**Frequency**
Screening was easy/good/fine	50
Screening was helpful	5
It was a relief to be screened	5
I felt my problem was being understood	5
I thought the screening questions were important and/or relevant	4
The screening raised my awareness of depression	4
Screening made me realise how I was coping	4
Screening was confronting/intimidating	3
Screening allowed me to reflect on my own mood	3
Screening highlighted the importance of being in contact with my nurse	3
The screening questions were not too personal	2
Screening was a little uncomfortable	2
Screening was a good idea	2
I wanted to know if I was at risk of PND	2
The screening questionnaire was administered in an unhelpful manner	2
